# Characterization of the complete chloroplast genome of *Rohdea wattii* (Asparagaceae)

**DOI:** 10.1080/23802359.2021.1967801

**Published:** 2021-08-25

**Authors:** Yifei Wang, Yancheng Zhang, Cailin Li, Bo Zhao

**Affiliations:** College of Pharmacy, Guilin Medical University, Guilin, China

**Keywords:** *Rohdea wattii*, chloroplast genome, phylogenetic relationship

## Abstract

As a kind of rare plant with important medicinal value and breeding value, *Rohdea wattii* usually grow in dark and damp places. In this study, we reported the complete chloroplast genome of *R. wattii*. We used a variety of bioinformatics analysis methods to analyze and visualize the chloroplast genome and phylogenetic relationships. A typical quadripartite structure was observed in the chloroplast genome of *R. wattii* with a genome size of 156,487 bp. The length of the large single-copy region (LSC) was 84,969 bp, which was the longest region among the four regions; the second longest was the Inverted repeat region (IR) with 26,451 bp in length; and the smallest was the small single-copy region (SSC) with 18,616 bp in length. The overall GC content of *R. wattii* chloroplast genome is 37.61%. In addition, a total of 132 genes were identified in the whole genome of *R. wattii*, which include 86 protein-coding genes, 38 tRNA genes, and eight rRNA genes. Finally, by constructing a phylogenetic tree to analyze the phylogeny of *R. wattii*, and it indicated that *R. wattii* and *Rohdea chinensis* are a close evolutionary relationship.

The *Rohdea wattii* is a perennial herb, which belongs to the family Asparagaceae (Yamashita and Tamura 2004; Nguyen et al. 2021). *Rohdea wattii* grows in dark and humid places and often appears in China, Vietnam, and other places (Tanaka 2010). As a kind of medicinal plant, *R. wattii* has long been used by the folk to treat sore throat, and it also has the medicinal effects of clearing heat and detoxification, dispelling blood stasis, and relieving pain (Shen et al. 2003). As a kind of plant with high medicinal value, *R. wattii* has been widely studied in the fields of pharmacology and phytochemistry (Yao et al. 2021), but the analysis of its genetic information is still scarce. We believe it to be an important yet underappreciated direction for the taxonomy and identification of this species. As the wild resources of *R. wattii* are gradually decreasing (Shen et al. 2003), it is more and more urgent to study its genetic and evolutionary relationship at the molecular level. In this study, we used Illumina high-throughput sequencing technique to obtain the complete chloroplast genome sequence of *R. wattii*, and analyzed its structural characteristics and phylogenetic relationship. These valuable results can facilitate us to efficiently formulate a strategy for the conservation of plant genetic resources, and minimize the risk of extinction of endangered plants.

In this study, the total genomic DNA of *R. wattii* was extracted from fresh leaves using a modified CTAB method (Doyle and Doyle 1987) and then quantified using a Nanodrop 2000 spectrophotometer. The samples were collected from Guangxi Institute of Botany, Chinese Academy of Sciences, Guilin, China (25°01′N, 110°17′E). Voucher specimen of *R. wattii* was deposited at the herbarium of Guangxi Institute of Botany (contact person: Bo Zhao, email: 2052886016@qq.com) under the voucher number ZB-RW202006003).

The sequencing was carried out on the Illumina Novaseq platform following the manufacturer’s protocol (Illumina, San Diego, CA, USA). After quality assessment, the chloroplast genome related reads were filtered by mapping all the raw reads to the reference chloroplast genome of *Rohdea chinensis* (MH356725; Zhou et al. 2018). The chloroplast genome annotation was performed through the online program CPGAVAS (Liu et al. 2012) and GeSeq (Tillich et al. 2017), and we manually corrected some necessary genes. Then the circular genome map was drawn by the OGDRAW program (Greiner et al. 2019) and the whole chloroplast genome of *R. wattii* was submitted to GenBank (MW822041). And the associated BioProject, SRA, and Bio-Sample numbers are PRJNA720828, SRR14203200, and SAMN18680948, respectively.

Like typical angiosperms (Wicke et al. 2011), the chloroplast genome of *R. wattii* possessed a characteristic quadripartite circular which consists of four regions: LSC, SSC and a pair of IRs. The total length of *R. wattii* chloroplast genome was 156,487 bp. The large single-copy region (LSC) in *R. wattii* was 84,969 bp and the small single-copy region (SSC) was 18,616 bp, which were separated by a pair of 26,451 bp inverted repeat regions (IRs). In the complete chloroplast genome, the contents of adenine (A), thymine (T), guanine (G), and cytosine (C) are 30.87%, 31.52%, 18.44%, and 19.17%, respectively. In addition, the GC content of IR region was 42.97%, which was the highest among the four regions; the second highest was LSC region, which was 35.61%; and the GC content in the SSC region was the lowest at 31.49%. A total of 132 genes were identified in the whole genome of *R. wattii*, including 86 protein-coding genes, 38 tRNA genes, and eight rRNA genes. Among them, there were 44 genes involved in photosynthesis, which included five genes for photosystem I, 15 genes for photosystem II, six genes for ATP synthase, 11 genes for NADH dehydrogenase, six genes for the cytochrome b/f complex, and one gene for the large chain of Rubisco. These genes related to photosynthesis are the key genes for studying the genetic evolution of *R. wattii*.

To study the phylogenetic relationship of *R. wattii*, a phylogenetic analysis was performed based on the complete chloroplast genome sequences of 19 species including the *R. wattii* chloroplast genome assembled in this study from Asparagaceae and two species from other families. The chloroplast genomes of 20 species were downloaded from the NCBI GenBank database. The sequences were aligned using MAFFT v7 (Mayor et al. 2000). Then we constructed the phylogenetic tree using Maximum Likelihood (ML) method by RAxML 7.0.4 (Stamatakis 2006) with 1000 replicates under the GTR + CAT model. *Lycoris anhuiensis* and *Lycoris chinensis* were designated as outgroup species. The phylogenetic tree revealed that *R. wattii* was a sister to *Rohdea chinensis*, and they contributed to a new evolutionary relationship to *R. wattii* in *Rohdea*, which is a genus of Asparagaceae in the order Asparagales (Figure 1). This analysis will help us to understand the evolutionary history of Asparagaceae species and provide new insights for better protection of endangered species.

**Figure 1.**
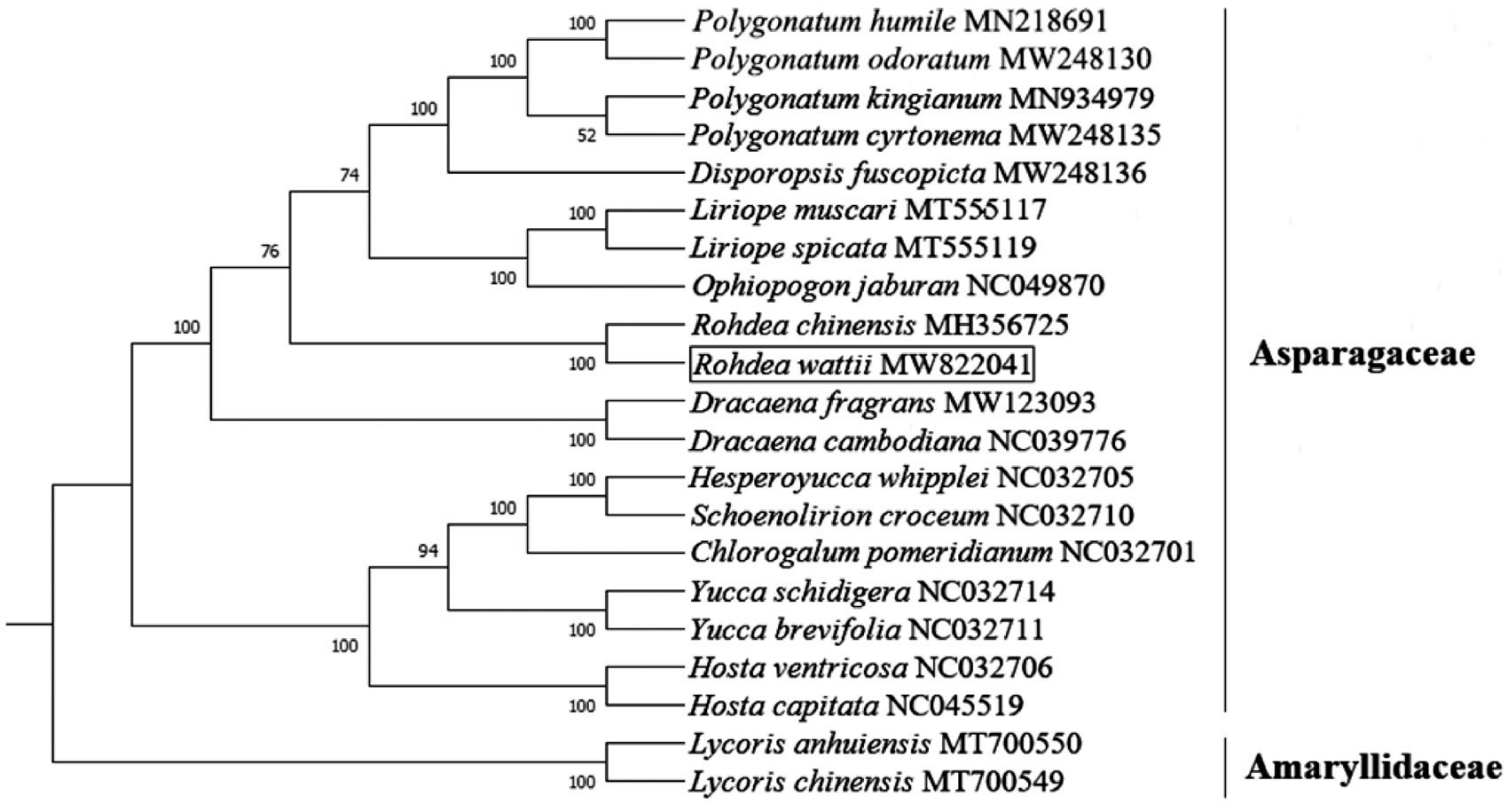
Phylogenetic tree construction using maximum likelihood based on the complete chloroplast genome sequences of *R. wattii* and other 18 species within Asparagaceae and two outgroup species (*Lycoris anhuiensis* and *Lycoris chinensis*). The numbers at the branches show bootstrap support values.

## Data Availability

The associated BioProject, SRA, and Bio-Sample numbers are PRJNA720828, SRR14203200, and SAMN18680948, respectively. The data that support the findings of this study are openly available in GenBank of NCBI at https://www.ncbi.nlm.nih.gov, reference number MW822041. Tree file of 21 species and genes for phylogenetic analysis were deposited at Figshare: https://doi.org/10.6084/m9.figshare.14609466.
